# Targeted approaches to improve tomato fruit taste

**DOI:** 10.1093/hr/uhac229

**Published:** 2022-10-11

**Authors:** Shouchuang Wang, Qi Qiang, Lijun Xiang, Alisdair R Fernie, Jun Yang

**Affiliations:** Sanya Nanfan Research Institute of Hainan University, Hainan Yazhou Bay Seed Laboratory, Sanya, 572025, China; College of Tropical Crops, Hainan University, Haikou, 570228, China; College of Tropical Crops, Hainan University, Haikou, 570228, China; College of Tropical Crops, Hainan University, Haikou, 570228, China; Max Planck Institute of Molecular Plant Physiology, Potsdam-Golm 14476, Germany; Sanya Nanfan Research Institute of Hainan University, Hainan Yazhou Bay Seed Laboratory, Sanya, 572025, China; College of Tropical Crops, Hainan University, Haikou, 570228, China

## Abstract

Tomato (*Solanum lycopersicum*) is the most valuable fruit and horticultural crop species worldwide. Compared with the fruits of their progenitors, those of modern tomato cultivars are, however, often described as having unsatisfactory taste or lacking flavor. The flavor of a tomato fruit arises from a complex mix of tastes and volatile metabolites, including sugars, acids, amino acids, and various volatiles. However, considerable differences in fruit flavor occur among tomato varieties, resulting in mixed consumer experiences. While tomato breeding has traditionally been driven by the desire for continual increases in yield and the introduction of traits that provide a long shelf-life, consumers are prepared to pay a reasonable premium for taste. Therefore, it is necessary to characterize preferences of tomato flavor and to define its underlying genetic basis. Here, we review recent conceptual and technological advances that have rendered this more feasible, including multi-omics-based QTL and association analyses, along with the use of trained testing panels, and machine learning approaches. This review proposes how the comprehensive datasets compiled to date could allow a precise rational design of tomato germplasm resources with improved organoleptic quality for the future.

## Introduction

**Figure 1:**
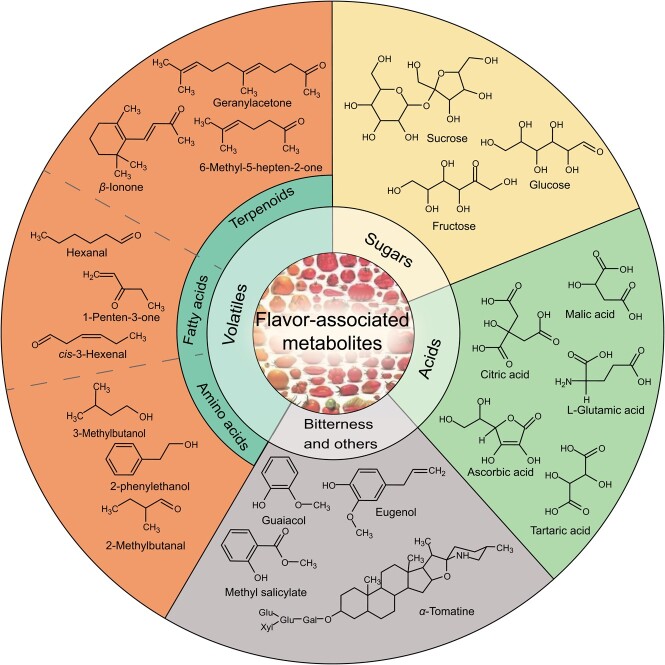
Classification of major metabolites associated with tomato flavor. Representative compounds related to flavor-related metabolites are shown, such as (1) sugars, (2) acids, (3) bitter compounds, and (4) volatiles derived from terpenes, fatty acids, and amino acids, each of which contribute to tomato flavor.

Tomato (*Solanum lycopersicum*) is the world’s most valuable fruit and horticultural crop species, contributes important nutrients to the human diet, and constitutes an important model system for plant biology research [1, 2]. Compared with the fruits of traditional or heirloom tomato cultivars, consumers often describe those of modern cultivars as tasteless [3, 4]. In the domestication and improvement of tomato, breeders have focused on key traits such as yield, disease resistance, fruit size, and attractive visual parameters as the primary goals of modern cultivars. As such, they have indirectly ignored the flavor and nutritional content of tomato fruits [5, 6]. Breeders have, however, partially focused on sugar and acid content as they are thought to significantly impact mouthfeel. However, previous studies have shown that fruit size is not significantly positively correlated with sugar content [7–9]. Moreover, changes in the metabolite profiles of cultivated tomato fruits over a long time are the main factors leading to the deterioration of flavor quality. With the improvement of people’s standard of living worldwide, consumers prefer delicious and high-quality fruits and are willing to pay a reasonable extra amount for enhanced quality [10, 11]. This has resulted in a step change of focus from yield-oriented to flavor-oriented breeding, with high-yielding flavorsome tomato fruits becoming the ultimate goal of breeders [12]. This increasing consumer interest has paralleled a rebirth of exploring tomato flavor at the scientific level, with its underlying genetic basis attracting increased amounts of attention from researchers in recent years [10, 11, 13, 14]. Tomato flavor arises not only from a complex mix of taste metabolites but also from the perception of volatile compounds. Tomato flavor is mainly composed of taste and volatile aromas, including sugars, acids, amino acids, vitamin C, and various volatiles [11, 15, 16] (Fig. 1). These compounds are responsible for the basic tastes of sweetness, sourness, bitterness, and umami, and aromas that may have a positive or negative effect on flavor [11, 17]. Studies have shown that >400 volatiles can be detected in tomato fruit; however, only a few volatile metabolites, such as aldehydes, esters, alcohols, ketones, lactones, and terpenoids, are known to have an important impact on tomato flavor [18]. Traditional flavor studies have typically focused on identifying a single compound, which makes studying the entire flavor profile and the relationships between and among stimuli (taste and aroma) impossible. Determining these interconnections is further complicated by flavor being a complex phenomenon influenced by both intrinsic and extrinsic factors, such as food shape and physiological and emotional factors [13, 18]. Indeed, flavor perception is multimodal, and smell is not only a stimulus but also a combination of taste, texture, and appearance, creating the desired flavor perception [18].

With the continuous development of metabolite detection technology and metabolic data mining tools based on gas chromatography–mass spectrometry (GC–MS), liquid chromatography-mass spectrometry (LC–MS), and nuclear magnetic resonance spectroscopy (NMR) technologies [19, 20], the analytical methods of ‘flavoromics’ based on metabolic profiling have gradually advanced. The term ‘flavoromics’ was coined by Reineccius at the 235th American Chemical Society (ACS) meeting in 2008 [21]. It describes the targeted and comprehensive analysis of all flavor-related metabolites based on metabolomics [21–23] and has been applied to various plant species, including coffee, grape, strawberry, canola, blueberry, and citrus [10, 24–28]. However, to date, by far the greatest research effort has been concentrated on tomato.

Here, we summarize the latest research related to tomato flavor and detail how machine learning has recently been applied to aid in this endeavor. We additionally highlight the applications and advantages of integrative multi-omics strategies in determining the genetic basis of tomato flavor. Finally, we present a metabolomics-based modern molecular marker breeding technique and suggest how it, along with machine learning-based approaches, can facilitate the genetic improvement of crop nutrition and flavor quality.

## Flavor-related metabolites of tomato

### Sugars and acids

As important historical breeding targets, sugar and organic acid contents are very important factors that directly affect the flavor of tomato fruits [29]. If these compounds are present at high concentrations and as long as they are maintained in a certain balance, sugars and acids can significantly improve the flavor of tomato fruits [29, 30]. Sugar is the most important factor influencing consumer preference, and there is a strong positive correlation between perceived sweetness and overall preference [18]. The fruit of cultivated tomato (*S. lycopersicum*) mainly contains hexoses derived from the hydrolysis of sucrose, such as fructose and glucose, and a small amount of sucrose, whereas the predominant form of sugar in the fruit of some wild varieties (e.g. *Solanum chmielewskii*) is sucrose [31, 32]. Compared with glucose, fructose is a better target metabolite because it is more than twice as sweet, so increasing the fructose concentration can more effectively improve the taste and quality of tomato fruits. Thus, deciphering the quantitative trait loci (QTL) for the fructose:glucose ratio was an important yet enigmatic task prior to the identification of the function of the Sugars Will Eventually Be Exported (SWEET) family of transporters [33]. Recently, a *SWEET* gene was identified as the causative gene underlying this tomato fruit trait [32].

In tomato fruit (*S. lycopersicum*), malic acid, citric acid, ascorbic acid, tartaric acid, and glutamic acid are the major organic acids, of which malic acid and citric acid are the predominant compounds affecting fruit flavor and palatability [34, 35]. Citric acid is the dominant organic acid at all stages of the tomato fruit life cycle, while malic acid is present at considerable levels in unripe green tomato fruits, and the malic acid content subsequently declines while fruits ripen [29, 36]. Increasing the malic acid concentration within the fruit results in significant changes in the tomato fruit’s starch metabolism and soluble solids content (SSC), affecting postharvest fruit softening [37]. Studies have confirmed that malic acid also plays an important role in enhancing the taste of sucrose during the consumption of tomato fruit [38]. Therefore, when consuming tomato fruits and developing new tomato varieties, a large number of consumers, as well as crop breeders in different regions, have consciously or unconsciously selected for a desirable malic acid content. However, the genotypic variation controlling malic acid levels during tomato domestication remains unknown [39, 40].

The changes in sugar and organic acid contents of tomato fruits are closely related to the ripeness of tomato fruits at different developmental stages [29]. The total content of sugar metabolites increases by 4% during ripening, with glucose mainly present in immature green fruits early in development, while fructose contents are usually higher than glucose contents in fully ripe fruits [32, 35]. However, after fruit ripening the total content of all sugar metabolites decreases [41]. The type and content of organic acids also change dynamically during fruit development, and research has shown that the content of acids increases during fruit maturation. As the main organic acid, citric acid is largely present in various stages of fruit development, but a large amount of malic acid can accumulate during the early stage of tomato fruit development. Similar to the trend of changes in sugar content, citric acid concentrations decrease with increasing ripeness after tomato ripening, while malic acid contents remain at relatively constant dynamic levels.

### Volatiles

Volatiles constitute essential parts of the flavor palate of tomato fruits. Like sugars and acids, volatiles provide the basis for good flavor, and removing them can significantly reduce the flavor intensity of tomato fruits [16, 42, 43]. According to a survey of consumer tastes, by enhancing the perception of sweetness, specific volatiles make tomato fruits taste sweeter than they are [11].

**Figure 2:**
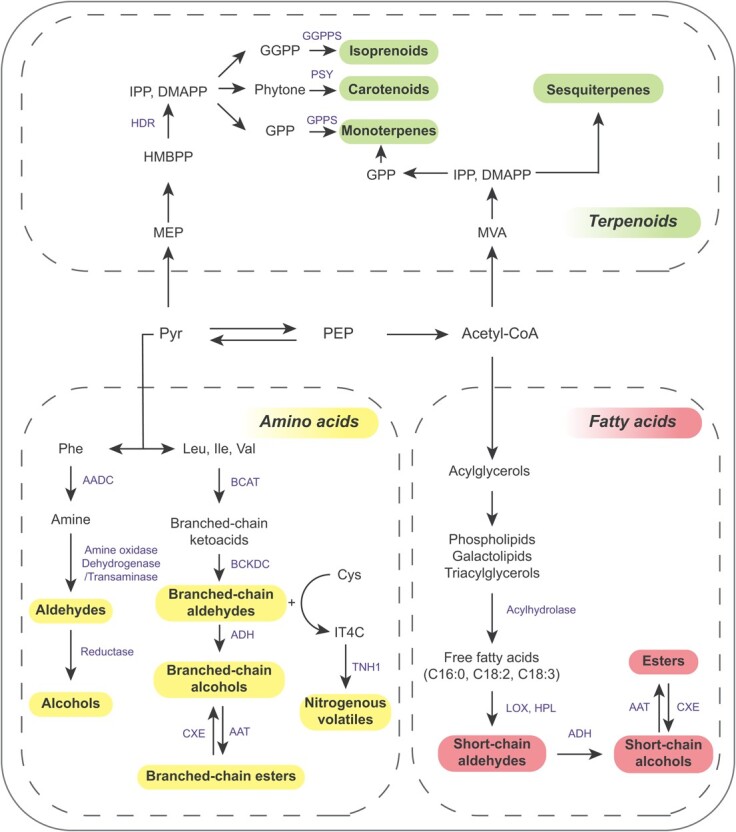
General summary of the volatile pathway in tomato. In this overview we summarize the primary volatiles derived from fatty acids (in red), amino acids (in yellow), and terpenoids (in green) that are synthesized from pyruvate (Pyr) and acetyl-CoA. The enzymes involved in this pathway are indicated in blue. The primary varieties of flavor-associated metabolites are indicated in bold. PEP, phosphoenolpyruvate; AADC, aromatic amino acid decarboxylase; BCKDC, branched-chain *α*-ketoacid decarboxylase; BCAT, branched-chain aminotransferase; CXE, carboxymethylesterase; AAT, alcohol aminotransferase; Phe, phenylalanine; Ile, isoleucine; Leu, leucine; Val, valine; Cys, cysteine; TNH, tetrahydrothiazolidine *N*-hydroxylase; IT4C, 2-isobutyltetrahydrothiazolidine-4-carboxylic acid; LOX, lipoxygenase; HPL, hydroperoxide lyase; ADH, alcohol dehydrogenase; HDR, 1-hydroxy-2-methyl-2-*E*-butenyl 4-diphosphate reductase; PSY, phytoene synthase; IPP, isopentenyl diphosphate; HMBPP, 1-hydroxy-2-methyl-2-*E*-butenyl 4-diphosphate; MEP, 2-*C*-methyl-d-erythritol-4-phosphate; GGPP, geranylgeranyl diphosphate; GPP, geranyl diphosphate; DMAPP, dimethylallyl diphosphate; MVA, mevalonate; GGPS, GPP synthase; GGPPS, geranylgeranyl diphosphate synthase.

Volatile compounds in tomato fruits are mainly derived from three biosynthesis pathways for which lipids, amino acids or terpenoid metabolites act as precursors (Fig. 2). The most abundant volatiles in tomato fruits are mainly short-chain aldehydes and alcohols, such as the lipid degradation products *cis-*3-hexenol and *cis-*3-hexenal, which afford tomato fruits a strong fresh green leaf aroma. During fruit ripening or tissue rupture, lipids are degraded by lipoxygenase (LOX) and hydroperoxide lyase (HPL) into short-chain aldehydes. Fatty acids such as linoleic acid (18:2) and linolenic acid (18:3) are first converted to hexanal and *cis-*3-hexenal, and then subsequently isomerized into *trans-*3-hexenal [44]. Aldehydes can be further transformed into alcohols via catalysis by alcohol dehydrogenase (ADH) and ultimately converted to esters by alcohol acetyltransferase (AAT1). Like *cis*-3-hexenyl acetate, 2-methylbutyl, and 3-methylbutyl acetate, acetates are generally not present in cultivated tomatoes but are higher in green-fruited wild tomato species [44]. Fatty acid-derived volatiles have an important impact on tomato flavor and are one of the important factors in consumers’ preference for tomato fruits. Indeed, the content of *Z*-4-decenal has a positive effect on consumer preference and flavor, while *E*,*E*-2,4-decadienal is also positively correlated with flavor intensity. Fatty acid-derived volatiles, such as 1-pentanol, *E*-3-hexen-1-ol, *E*-2-heptenal, and 1-octen-3-one, also contribute significantly to consumer preference and flavor intensity [7, 45].

Biosynthesis of amino acids is also an important source of tomato volatiles, including l-phenylalanine, l-leucine, and l-isoleucine. These amino acids form a series of typical volatiles, such as 2- and 3-methylbutanal and 3-methylbutanol, that are essential flavor components in tomato fruit. Branched-chain amino acids are broken down into different branched-chain keto acids by the action of branched-chain amino acid transaminase (BCAT) [44, 46]. Studies have shown that in transgenic tomato plants overexpressing *SlBCAT1*, the contents of branched-chain-derived volatiles, such as 2-methyl or 3-methylbutanal, 2-methyl or 3-methylbutanol, isovaleronitrile, isobutyl acetate, and 2-isobutylthiazole, are increased by 1.3- to 2.5- fold [47]. Phenylalanine is decarboxylated by aromatic amino acid decarboxylase (AADC) to form phenethylamine, which can be converted to 2-phenylacetaldehyde by amine oxidase, dehydrogenase, or transaminase. Subsequently, 2-phenylacetaldehyde reductase can be converted to 2-phenylethanol [48–50]. In addition, eugenol, a phenylpropene compound with a clove-like aroma derived from phenylalanine, is found in low levels in tomatoes [51]. Moreover, methyl salicylate, a phenylalanine-derived compound, is also thought to be an essential contributor to tomato flavor, presumably resulting from the methylation of salicylic acid [52, 53]. Furthermore, cysteine has been proved to provide the nitrogen atom for some nitrogenous volatile metabolites which are synthesized by a tetrahydrothiazolidine *N*-hydroxylase (SlTNH1) [54]. Relatedly, GDSL esterase/lipase enzymes were proven to be significantly associated with asparagine, *γ*-aminobutyrate (GABA), glutamine, and threonine [14].

Terpenoid volatiles, which are synthesized by way of the mevalonate (MVA) and methylerythritol phosphate (MEP) pathways, are derived from isopentenyl diphosphate (IPP) or its isomer dimethylallyl diphosphate (DMAPP). The terpenoid volatiles in tomato may be the product of synthesis following the direct catalysis of monoterpene and sesquiterpene synthases, or the decomposition of larger terpenoids such as diterpenes, triterpenes, and tetraterpenoids, such as plastid carotenoid pigments [52, 55, 56]. Besides monoterpenes and sesquiterpenes, norisoprene, which comes from the degradation of larger terpenoid carotenoids, has a significant effect on consumer perception of tomato fruit aroma [1, 52, 56, 57]. Monoterpenes have additionally been shown to be one of the most important contributors to many floral and fruit aromas of plants, and their biosynthetic precursor is geranyl diphosphate, but mature fruits contain only trace amounts of monoterpenes. The most common monoterpene found in tomatoes is citral, which is a mixture of *cis-* and *trans-*acyclic monoterpene aldehyde isomers, along with neral and geranial, which give tomato fruits their pleasant lemon-like aroma. Linalool, an acyclic monoterpene alcohol found in tomato fruit, presents a fragrance reminiscent of flowers. In addition, members of the tomato terpene synthase family (TPS) function in the synthesis of several terpenoid volatiles, such as *β*-myrcene, limonene, linalool, 1,8-cineole, *β*-phellandrene, and nerolidol [58].

There is also a particularly important class of carotenoid-derived volatiles, norisoprene (carotenoid) metabolites, which result directly from the oxidative cleavage of carotenoids such as *β*-carotene and neoxanthin [55]. The accumulation of norpentadienes in fruits is usually very low, but these compounds play an important role in the overall human perception of tomato flavor. Experiments involving volatiles showed a decrease in *β*-ionone and geranylacetone contents in tomatoes, which were affected by the carotenoid cleavage dioxygenase (*LeCCD1*) gene when these important flavor-related volatiles were formed *in vivo* [56].

### Bitter and other compounds

Common modern cultivars of tomato are generally devoid of bitterness because they are deemed undesirable flavors for consumers. Therefore, modern cultivars of tomatoes have undergone a robust negative selection for bitter tastes in tomato breeding [7]. Steroidal alkaloids (SAs) and their glycosylated forms (SGAs) are a class of nitrogen-containing secondary plant metabolites commonly found in nightshade plants, including potato (*Solanum tuberosum*), tomato (*S. lycopersicum*), and eggplant (*Solanum melongena*) [58, 59]. A common precursor for SGA biosynthesis is cholesterol, which undergoes various metabolic modification reactions such as hydroxylation, oxidation, transamination, and glycosylation, resulting in rich metabolic diversity in terms of the molecular structure of this natural product [60]. SAs and SGAs are specialized metabolites that act as chemical barriers against various pests and pathogens. SAs are considered harmful antinutritional factors in humans and animals because they negatively affect the digestion and absorption of nutrients in food and may even lead to toxicity [61]. SGA in tomato fruit produces an unpleasant bitter taste, which is significantly negatively correlated with good flavor. In tomato, the number and total content of SGAs in wild plants (*Solanum pimpinellifolium*) were shown to be significantly higher than in cultivated plants; although the SGA content was generally lower than that in potato, tomato still contained ~150 SGAs [62]. Dehydrotomatine and *α*-tomatine, which are less glycosylated, predominate in immature fruits, while the most abundant SGA in mature fruit is esculin. During the ripening process of tomato fruits, tannol is converted to hydrolyzed tomatine under the action of cytochrome P450 and then undergoes a series of glycosylation and hydroxylation reactions to form aescin [60]. Tomatine undergoes multiple glycosylations, the results of which can increase the resistance of plants to pathogens and reduce the toxicity and bitterness of tomatine itself. While the total SGA content remains unchanged during fruit ripening, a dynamic transformation of the *α*-tomatine pool occurs on the transition from immature green fruits to ripe tomato fruits [63].

In addition to glycoside sterol alkaloids, flavanone derivatives are also bitter in tomato fruits [64]. Flavonoids accumulate mainly in the tomato fruit peel, and only trace amounts of flavonoids can be found in the pulp [65]. The main flavonoids in the tomato fruit peel are naringenin-chalcone and rutin (quercetin-rutinoside) [66]. Naringin, the main flavanone glycoside extracted from grapefruits, is one of the important metabolites responsible for the bitter taste of citrus [67]. This flavonoid is also found in the peel of tomatoes. However, because naringin levels are low in the tomato fruit peel, they usually do not contribute significantly to tomato flavor [68]. Nonetheless, naringin is highly important since experiments have shown that this compound can reduce inflammation, protect mice from various diseases, and promote heart health [69].

## Discovery and characterization of flavor-related metabolites based on metabolomic technology

The metabolome determines the nutritional value, flavor composition, and color of tomato, so the detection and identification of metabolites is the major route for studying tomato flavor [6, 18]. With the continuous advancement of detection technology, metabolomics based on GC–MS, LC–MS, and NMR technologies have extensively promoted the analysis of the tomato metabolome [4, 50, 70].

First, the types of metabolites that can be analyzed by LC–MS and GC–MS are different. Normally, semipolar metabolites are detected through LC–MS. Recently, a high-resolution spatiotemporal metabolome was determined via LC–MS in order to explore the differences and characteristics of metabolites among 20 major tomato tissues and growth stages. More than 540 metabolites were detected, including SA and flavonoids; this study provided new insights into the dynamic features of tomato metabolism [71]. However, analysis of lipids, volatile organic compounds, and derivatized molecules often uses GC–MS. Some representative volatiles, such as phenylpropanoid volatiles and short-chain esters, can be released from tomato fruits after ripening. Tikunov *et al*. analyzed the metabolite profiles of distinctly different tomato cultivars via GC–MS and suggested that, upon fruit tissue disruption, the presence of phenylpropanoid volatiles (methyl salicylate, guaiacol, and eugenol) in ripening fruits resulted in unpleasant flavors [72, 73]. The same measurement was carried out on volatile esters to prove that the divergence of alcohol acyltransferase impacts fruit branched-chain ester composition [16]. Metabolite characteristics can also be thoroughly defined via NMR, which is advantageous by allowing the determination of metabolite levels in a non-invasive manner. Using this spectroscopic technique, van Schadewijk *et al*. investigated large-scale metabolites and recorded how the levels of phenylpropanoids, monoterpenes, and tocopherols increased before grape berries matured [74]. In recent years, chemical exchange saturation transfer (CEST) MRI has been used to map tomato metabolites, allowing the exploration of features of specific flavor-related metabolite distributions in fruit tissues. In these studies, it was shown that glucose and fructose accumulated mostly in the locular tissue, whereas glutamic acid and glutamine were located largely in the columella [70].

Using the above methods to identify and validate compounds associated with consumer preference is an integral part of flavor research [7]. Flavor should not be viewed only in hedonistic terms, however, since tomato is a highly nutritious food containing high amounts of vitamins, especially carotenoids and vitamin C, as well as a wide range of phytonutrients, especially phenylpropanoids [75, 76]; increasing the flavor of tomato and hence its consumption would provide considerable health benefits owing to the proven bioactive functions of taste-neutral components of the fruits [77].

Flavoromics is a method that integrates metabolomics techniques and human sensory evaluation for studying all flavor-related metabolites. This method combines subjective studies involving flavor fingerprinting, screening via metabolite analysis of flavors, and chemometrics research methods to study the quality of products and the authenticity of claims made for products of specific quality [78, 79]. Combining factors such as smell, taste, mouthfeel, and appearance is necessary to identify and quantify some of the specific flavor-related compounds that are associated with subjective perception [7]. Recent work adopting sensory testing to rate multiple sensory attributes revealed 33 metabolites associated with consumer preference and 37 significantly associated with flavor intensity. Malic acid, salicylaldehyde, butyl or hexyl acetate, and isoprenyl or isobutyl acetate were proven to be significantly negatively correlated with flavor. Among these compounds, carotenoid volatiles such as geranylacetone, 6-methyl-5-hepten-2-one (MHO), *β*-ionone, and geranial were found to directly affect consumer preference. In addition, the use of e-nose- and e-tongue-based devices has become a standard approach for measuring volatile and non-volatile compounds [80–82]. Detection of tomato flavor-related metabolites by e-noses following different postharvest handling treatments and after several days of chilling storage has been reported, the findings of which suggest significant changes in the aroma of tomato fruits in response to these storage and harvest strategies [21, 81]. Moreover, the e-tongue can be used to explore the soluble compounds of tomato fruits to accurately determine their composition, and sensory properties of the tested tomato samples can be further applied for monitoring the authenticity of processed products [82].

## Integrative omics analysis can reveal the genetic basis for tomato flavor formation

Based on the continuous development of low-cost, high-throughput technologies, tomato flavoromics research has also entered the era of big data by way of multi-omics integrated analysis, mainly including genomes, transcriptomes, proteomes, and metabolomes [83–86]. Next-generation sequencing technologies facilitate the acquisition of high-quality reference genomes and provide more readily available information of population variants, thereby facilitating the efficient and precise identification of genetic variation and allowing the construction of high-density physical and genetic linkage maps [34, 43, 87].

Comprehensive transcriptomic and metabolomic analysis is an effective method to mine genes related to the synthesis and regulation of metabolic pathways and is a powerful tool for analyzing the genetic basis of metabolic pathways. Comparative co-expression analysis between tomato and potato revealed 10 glycoalkaloid metabolism (*GAME*) genes involved in SGA biosynthesis, of which six are present in a gene cluster on chromosome 7 [60]. In addition, three other genes encoding cytochrome P450s (P450s), namely, *GAME7*, *GAME8a*, and *GAME8b*, are also involved in SGA biosynthesis despite not being within these clusters [88]. Under the action of these *GAME* genes, cholesterol undergoes hydroxylation, oxidation, transamination, and glycosylation before SGAs are ultimately produced. Thus, a clear pathway for the biosynthesis of Solanaceae SGAs from cholesterol precursors to SGAs has been proposed [61]. Subsequent metabolomic studies on a range of wild tomato species were able to clarify these pathways via further refinements [89]. More recently GAME9, an AP2/ERF transcription factor, was found to regulate steroidal alkaloid biosynthesis in Solanaceae by controlling SGA biosynthesis and several upstream mevalonate and cholesterol precursor pathway genes [90].

Linkage maps and genome-wide association studies (GWAS) for studying the genetic basis of natural variation within a metabolome are excellent tools for unearthing superior tomato flavor-related alleles [92, 92]. The use of these complementary approaches has resulted in the identification of a large number of genes related to flavor (Table 1). For example, the sugar transporter *STP11* (Solyc06g054270) was identified by a GWAS, and its allelic variation affects changes in SSC contents in tomato fruits [93]. The natural variants of the Solyc07g055840 and Solyc12g008430 genes, which encode citrate synthase and malic enzyme, respectively, were significantly associated with the contents of citric acid and malic acid in a tomato population [14]. Similarly, the genetic basis of a range of desirable or deleterious aspects of flavor was revealed. Among these aspects, *LIP8* (Solyc09g091050) is an essential factor that participates in the synthesis of fatty acid-derived metabolites, such as *E*-2-hexen-1-ol and hexyl alcohol, while phytoene synthase (Solyc03g031860) and 3-methyl-2-oxobutanoate dehydrogenase (Solyc06g059850) play important roles in 6-methyl-5-hepten-2-one and 2-methyl-1-butanol production, respectively [7, 14, 94, 95].

**Table 1:** List of related genes that are related to flavor and used as part of the GWAS and linkage mapping analysis of tomato

**Primary pathway**	**Metabolites**	**Related genes**
Sugars	Fructose, glucose	*Lin5*, *STP11*, *SWEET* [7, 14, 32]
	Citric acid	Citrate synthase [14]
Acids	Malic acid	*SlALMT*, *TFM6*, Malic enzyme [14, 39]
Alkaloids	Steroidal alkaloids	*GAME1*, *GAME2*, *GAME5–9*, *GAME11*, *GAME12*, *GAME17*, *GAME18*, *GAME25*, *GAME31* [59, 60, 62, 88, 108]
	Proline	Serine incorporator [14]
	2-Isobutylthiazole, 3-methylbutyraldehyde oxime, 1-nitro-3-methylbutane, 3-methylbutanenitrile	*SlTNH1* [54]
	Asparagine, GABA, glutamine, threonine	*GDSL* esterase/lipase [14]
Amino acids	2-Methyl-1-butanol, 3-methyl-1-butanol	*BCKDC*, *SlBCAT1*, *SlBCAT2*, *AAT1* [16, 109]
	2-Phenylethanol	*LeAADC1A*, *LeAADC1B*, *LeAADC2*, *PPEAT*, *FLORAL4* [7,43, 110]
	Phenylacetaldehyde	*LeAADC1A*, *LeAADC1B*, *LeAADC2*, *GT* [7, 94]
	Guaiacol, methyl salicylate	*E8* [7]
Phenylalanines	Methyl salicylate	*SlSAMT* [53]
Carotenoids	6-Methyl-5-hepten-2-one, geranylacetone	Phytoene synthase [7]
	Geraniol	*GES* [111]
Monoterpenes	*β*-Myrcene, limonene	*TPS7* [58]
Sesquiterpenes	Linalool, 1,8-cineole, *β*-phellandrene, nerolidol	*TPS3*, *TPS5*, *TPS20*, *TPS37*, *TPS39* [58]
Terpenoids	Geranylacetone, pseudoionone, *β*-ionone	*LeCCD1A*, *LeCCD1B* [56]
	1-Octen-3-ol	*SlFAD7* [94]
	1-Pentanol	*SlFAD7*, *Sl-LIP8* [94, 95]
	1-Penten-3-ol, *E*-2-hexen-1-ol, hexyl alcohol, *Z*-3-hexen-1-ol,	*Sl-LIP8* [95]
	C5 or C6 volatiles	*LOXH1*, *LoxF*, *TomloxC* [112 –114]
	Alcohols, acetic acid	*SlCXE1*, *SlCXE5* [115]
Fatty acids	Hexanol, *Z*-3-hexenol	*ADH2* [116]

Another candidate QTL in tomato plants was found to harbor the gene *E8* (Solyc09g089580), which regulates fruit ripening because its product is a putative negative regulator of ethylene biosynthesis and is linked to the volatiles guaiacol and methyl salicylate, thereby leading to ‘smoky’ or ‘medicinal’ flavors [7, 87]. More than 50 QTLs affecting volatile levels have been identified recently [6, 96]. For example, the relation between a biosynthetic pathway of phenylalanine-derived volatiles in tomato and a series of aromatic l-amino acid decarboxylases (*LeAADC1A*, *LeAADC1B*, and *LeAADC2*) was revealed, demonstrating that AADCs participate in the synthesis of 2-phenylethanol and 2-phenylacetaldehyde [50]. Furthermore, Tikunov *et al*. also reported a QTL that affects the levels of phenylalanine-derived volatiles (PHEVs) in tomato. At the same time, the candidate gene *FLORAL4* was finely mapped to the PHEV locus [43]. A meta-analysis involving a GWAS of 775 tomato accessions and 2 316 117 single-nucleotide polymorphisms (SNPs) revealed 305 significant associations with sugar, acid, amino acid, and flavor-related volatile contents, 211 of which were novel. Among the 211 associated loci, 37 promising candidate genes with functional annotations associated with pathways of flavor-related compounds were identified. Thus, sugar content has not been under strict selection throughout tomato domestication and improvement. Indeed, modern cultivars have lost most of the allelic diversity through which sugar, acid, and volatile contents in ancestral varieties of this species are determined [22]. However, the strong positive correlation between the aggregation of multiple elite alleles and sugar content provides clues and resources for breeding new varieties with increased sugar contents [7]. Thus, it seems reasonable to conclude that in future breeding programs the combined application of multiple alleles for excellent traits could result in the production of new delicious tomato varieties.

According to the results of a GWAS of malate content, a single region on chromosome 6 was found to contain multiple SNPs that positively correlated with malic acid levels [39]. Two candidate Al-ACTIVATED MALATE TRANSPORTER (*ALMT*) genes, Solyc06g072910 and Solyc06g072920, were included in the respective chromosomal intervals and were discovered to correspond to a single gene (*Sl-ALMT9*) separated by a 3708-bp intron [85]. *Sl-ALMT9* highly regulates fruit malate accumulation and functions as a malate efflux transporter in tomato roots.

To reveal why modern cultivated tomato fruits lost their flavor, 398 tomato germplasm resources, including modern, heirloom, and wild accessions, were recently studied using metabolomic methods, and 101 different accessions were identified through consumer sensory evaluations [7]. The study revealed 33 and 37 chemicals that were significantly correlated with consumer liking and flavor intensity, respectively, of which 28 were significantly associated with both overall liking and flavor intensity. It was also found that, compared with their wild relatives, modern commercial varieties contained significantly lower levels of many essential flavor-related compounds [60]. A metabolite-based GWAS (mGWAS) was performed on the contents of 27 volatiles, total soluble solids, glucose, fructose, citric acid, and malic acid in ripe fruits, and a total of 251 associated signals were found for 20 traits, namely, four non-volatile- and 15 volatile-associated flavor chemicals [60]. On the basis of these results, the negative relationship between tomato fruit weight and sugar content may be related to the loss and improvement of high sugar-related alleles during domestication when larger fruits were selected [60]. The study of metabolic changes in domestication has also recently received considerable amounts of attention from researchers [7, 97–99]. Ripe tomato fruits have accumulated considerably lower amounts of antinutritional steroidal glycoalkaloids throughout the long period of domestication [86]. Metabolic restructuring during domestication has additionally resulted in marked changes in the degree of accumulation of metabolites such as amino acids and unsaturated fatty acids [99].

The types and contents of several flavor substances in tomato fruit were revealed to have been consciously or unconsciously selected by breeders. By integrating genomic, transcriptomic, and metabolomic data, researchers constructed a large dataset of gene-SNP metabolites, and 3526 mGWAS signals, 2566 *cis-*eQTLs, 93 587 *trans-*eQTLs, and 232 934 expression–metabolite correlations were identified [88]. Metabolomic analysis of different tomato subpopulations indicated that the SGAs determining tomato bitterness and toxicity were subject to substantial negative selection. A newly identified gene cluster was found on chromosome 10, consisting of one acetyltransferase, one cytochrome P450, one acetyl-CoA dehydrogenase, and seven UDP-glucosyltransferase genes, all of which may be involved in the biosynthesis of SGAs. A key observation of this massive study was that, in focusing on key breeding traits such as yield and disease resistance, breeders have inadvertently lost the elite alleles that determine tomato nutrition and flavor. However, there remain considerable flavor differences among tomato varieties that are difficult for us to currently define in terms of specific flavor-related metabolite composition. These findings call for considerably more research to comprehensively understand flavor.

**Figure 3:**
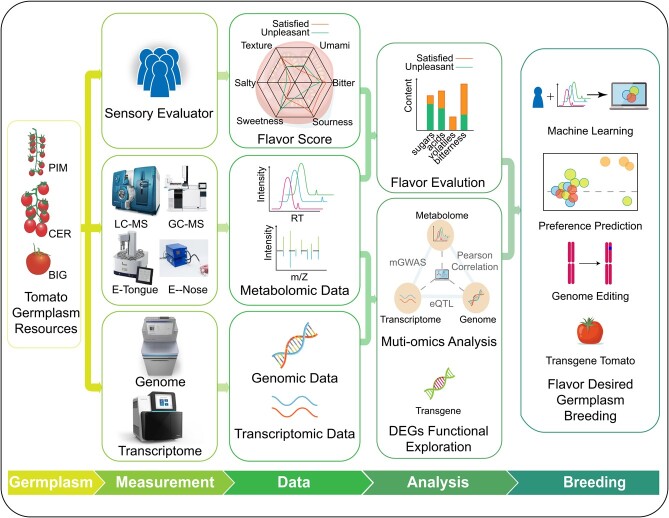
Strategy of flavor exploration based on the results of a multi-omics analysis. This flow of flavor exploration can be divided into five parts: tomato germplasm resource collection, sample measurement and sensory evaluation, data acquisition, comprehensive data analysis, and application. These tomato germplasm resources are subjected to several metabolic- and genetic-related measurements after collection and evaluated in multiple sensory attributes by consumers. Then multi-omics data are comprehensively analyzed to confirm flavor-related metabolites and molecular biosynthesis mechanisms. Finally, based on big data from multi-omics, a machine learning model was constructed to predict the flavor preferences of consumers. Related genes or loci that influence the flavor-related metabolite pathways can be edited in the tomato genome to obtain novel tomato accessions. DEGs, differentially expressed genes.

## Multi-omics-based sensory evaluation and machine learning for delicious tomato breeding

Tomato flavor is an important agronomic trait affected by multiple factors, including both objective factors, such as metabolites in the fruits, and subjective factors, such as consumers’ perception of taste and smell, and psychological effects [102, 103]. Although fruit flavor is a comprehensive breeding target trait, only individual and relatively simple methods have been used in past breeding processes to quantify flavor quality, including fruit acidity, sweetness, firmness, SSC content, and taste-test results [10, 104]. Therefore, systematic capture and accurate assessment of fruit flavor traits is a prerequisite for breeding and developing tomato varieties with improved flavor properties. In breeding, the evaluation of flavor is currently carried out by consumer sensory panels or the breeders themselves. Because the evaluators’ standards are not unified, on-site evaluation is usually highly subjective and prone to error; it can only represent the sensory preferences of a oneself or a few people [10]. In contrast, sensory panels based on large-scale populations are more scientific, objective, and accurate. However, quantifying sensory perception is time-consuming and labor-intensive, and it is difficult to achieve rapid high-throughput analysis. Taken together, these facts explain why breeders have struggled to accurately evaluate tomato flavor [10]. Low-cost and efficient methods for the detection of flavor compounds would greatly benefit the breeding process, and metabolomic methods can be used to detect more metabolites with small but non-negligible effects to achieve higher selection accuracy. In recent years, widely targeted metabolomics methods based on LC–MS and GC–MS have been developed [105, 106]. These methods have the advantages of low cost, having high throughput, and displaying ultrahigh sensitivity and the ability to cover different metabolites. They have therefore been widely used in the metabolic profiling of rice, maize, wheat, tomato, and a range of medicinal plants [88, 101, 105–107]. The main prerequisite for tomato flavor perception is the class and contents of metabolites, which mainly include various sugars, acids, amino acids, lipids, and volatiles, the contents of which are influenced by genetic and environmental factors [4, 32, 34]. The development and application of metabolomics have enabled large-scale analysis of natural populations comprising wild tomato fruits and heirlooms, and when more genetic variation within the gene pool is available, metabolomics can be used to accurately characterize flavor profiles in the early stages of breeding, avoiding the loss of high-quality flavor genotypes during breeding [108].

With the continuous development of detection technology and the accumulation of a large amount of metabolomics-related data, researchers are increasingly interested in the application of artificial intelligence technology to metabolomic big data analysis [108]. However, there has been little progress in tomato fruit flavor research at the metabolic level involving in-depth algorithms and novel machine learning models. To improve the efficiency and throughput of flavor phenotypic identification and analysis methods, the application of statistical and machine learning models can enable the prediction of consumer sensory panel rating scores based on the metabolic composition of fruits. When the types and contents of metabolites in fruits and their corresponding consumer perception evaluation scores are first quantified, a metabolite-based predictive model of consumer taste preferences can be created [7, 11]. Then, specific metabolites that constitute consumer taste preferences can be screened by utilizing a trained model. Prediction using metabolomics is challenging due to the complex correlations among various metabolites. Therefore, it requires a large sensory panel involving various flavor assessment scenarios to tune and calibrate a prediction model. To explore which metabolites in tomato fruits have an important impact on consumers’ flavor perception, the model was trained using metabolome and sensor panel data from 209 tomato samples, and different modeling methods were selected for further inference [10]. The findings of this study suggested that glucose and fructose are the most important sensory perception enhancers for the sweetness of tomato fruits. Gradient boosting machine learning approaches estimated that 1-penten-3-one and 2-phenylethanol play an important role in consumers’ perception of sweetness, while the Bayes A model predicted that the volatiles *E*-2-pentenal and 4-carene have significant effects on sweetness enhancement. Predictive analysis indicated that *E*-2-pentenal is also an important contributor to the overall flavor intensity and umami of tomato. When combined with genomics, artificial intelligence can be anticipated to identify both complex and subtle molecular regulations underlying flavor on the basis of massive datasets [109] (Fig. 3).

## Conclusions and future directions

The focus of tomato breeding has expanded from traditional agronomic traits such as yield and disease resistance to nutritional fortification and flavor improvement. With the rapid development of genomics, transcriptomics,, and metabolomics, the metabolic pathways of the main compounds that affect the formation of tomato flavor have been analyzed individually, and considerable progress has been made in the screening and functional verification of genes involved in the synthesis and regulation of these metabolites. Notably, improvement in tomato flavor may require the coordinated regulation of multiple biosynthetic pathways, and the optimal concentrations and stoichiometric ratios of various metabolites have not been fully established. Therefore, it seems likely that identifying new flavor regulation-related genes in other species would help advance the genetic improvement of tomato flavor. With the continuous development of high-throughput omics technologies and the continuous accumulation of multi-omics data such as genome, transcriptome, metabolome, and phenotype, omics resources and molecular toolboxes are rapidly expanding (Fig. 3). At the same time, group-based sensory panel and machine learning model training are also generating large amounts of data. Establishing a scientific evaluation process and an objective flavor perception prediction model will become the basis for accurate flavor omics research. In this vein, integrating multi-omics resources and artificial intelligence algorithms, with the use of gene editing methods, may allow the aggregation of important traits such as flavor, yield, and disease resistance, thereby greatly promoting the cultivation of new varieties of delicious tomatoes.

## Acknowledgements

We apologize to those authors whose work could not be discussed due to space limitations. This work was supported by the Hainan Province Science and Technology Special Fund (ZDYF2022XDNY144), the National Natural Science Foundation of China (No. 32100212), the National Key R&D Program of China (2021YFA0909600), the Young Elite Scientists Sponsorship Program by CAST (No. 2019QNRC001), the Hainan Provincial Academician Innovation Platform Project (No. HD-YSZX-202004), the Hainan University Startup Fund [Nos. KYQD (ZR) 1916 and KYQD (ZR) 21025] and the Innovation Project of Postgraduates of Hainan Province (No. Qhys2021-171).

## Author contributions

S-C.W. and J.Y. conceptualized and prepared the review and wrote the original draft. J.Y., Q.Q., L.-J.X., S.-C.W., and A.R.F. conducted the literature review and edited the manuscript. Q.Q. constructed the figures. S.-C.W. was responsible for the conceptualization and funding acquisition.

## Conflict of interest

The authors declare that they have no conflict of interest.
